# Imaging in non-cystic fibrosis bronchiectasis and current limitations

**DOI:** 10.1259/bjro.20210026

**Published:** 2021-07-29

**Authors:** Roberta Eufrasia Ledda, Maurizio Balbi, Francesca Milone, Andrea Ciuni, Mario Silva, Nicola Sverzellati, Gianluca Milanese

**Affiliations:** 1 Scienze Radiologiche, Department of Medicine and Surgery (DiMeC), University of Parma, Parma, Italy

## Abstract

Non-cystic fibrosis bronchiectasis represents a heterogenous spectrum of disorders characterised by an abnormal and permanent dilatation of the bronchial tree associated with respiratory symptoms. To date, diagnosis relies on computed tomography (CT) evidence of dilated airways. Nevertheless, definite radiological criteria and standardised CT protocols are still to be defined. Although largely used, current radiological scoring systems have shown substantial drawbacks, mostly failing to correlate morphological abnormalities with clinical and prognostic data. In limited cases, bronchiectasis morphology and distribution, along with associated CT features, enable radiologists to confidently suggest an underlying cause. Quantitative imaging analyses have shown a potential to overcome the limitations of the current radiological criteria, but their application is still limited to a research setting.

In the present review, we discuss the role of imaging and its current limitations in non-cystic fibrosis bronchiectasis. The potential of automatic quantitative approaches and artificial intelligence in such a context will be also mentioned.

## Introduction

The term *bronchiectasis* refers to a clinicoradiological entity characterised by an abnormal and permanent dilatation of the bronchial tree associated with respiratory symptoms, including productive cough and dyspnoea.^1^ A rather rough aetiological classification distinguishes two main groups of bronchiectasis: cystic fibrosis (CF) bronchiectasis and non-CF bronchiectasis. The latter recognises a broad spectrum of causes and associations, ranging from chronic obstructive pulmonary disease to allergic bronchopulmonary aspergillosis (ABPA) and tuberculous-associated lung destruction.^2–4^ Nevertheless, a non-negligible proportion of patients remains diagnosed with idiopathic bronchiectasis despite extensive testing, facing a potentially worse prognosis as compared to those affected by a known, treatable disease.^4,5^

Due to the increasingly larger use of computed tomography (CT)^6,7^ and greater awareness among physicians of its clinical and prognostic implications (*e.g.* hospitalisation for severe exacerbations, increased mortality rate, etc), the prevalence of non-CF bronchiectasis has been progressively rising over the last decades,^8,9^ reaching up to 566 per 100,000 inhabitants in some patient cohorts.^10,11^ The diagnosis of such an entity strongly relies on imaging, which is essential to identify abnormally widened airways.^12,13^ Additionally, imaging allows to: (i) quantify the airway dilatation; (ii) suggest the aetiology (in a few cases though), and (iii) evaluate the course of disease.^14^ Although chest X-ray (CXR) has been indicated as the first-line imaging modality for assessing bronchiectasis, it has substantially lower sensitivity and specificity compared to chest CT, which now represents the diagnostic reference standard.^15,16^ The diagnosis of bronchiectasis on CT, however, is based on the visual assessment of bronchial dilatation and is subjected to radiologist expertise as standardised diagnostic criteria are still to be defined.^17,18^ Several visual scoring systems have been used to assess the severity of bronchiectasis, showing substantial limitations.^19–21^ More recently, quantitative post-processing methods have been developed to objectively define bronchiectasis and quantify disease severity, but their application is mostly limited to a research setting.^18^

In the present review article, we discuss the role of imaging and its current limitations in non-CF bronchiectasis, hereafter named bronchiectasis. The potential of automatic quantitative approaches and artificial intelligence in such context will be also mentioned.

## Radiological definition of bronchiectasis

### Chest radiography

Although CXR is considered the first-line imaging modality in the diagnosis of bronchiectasis, it has limited sensitivity in detecting airway dilatation. Even when pronounced, the radiographic features of bronchiectasis are usually non-specific, and rarely provide direct evidence of bronchial dilatation.^22^ These features include increased pulmonary markings and, in case of cystic bronchiectasis, thin-walled cysts with or without air-fluid levels.^22,23^ Increased lung markings are related to thick-walled bronchiectasis that fails to end in a tapered fashion towards the lung periphery. When such bands run parallel to each other result in the so-called “tram-track” (i.*e.* resembling a railway). If the dilated bronchus and the accompanying pulmonary artery branch are seen in cross-section, the “signet ring” sign can be appreciated.^24^ However, even other disorders such as bronchitis without bronchial dilation or purely vascular disorders may manifest with similar radiographic features.^22^ Other recognised radiographic features of bronchiectasis include tubular and branching opacities caused by mucus plugging within the bronchial lumen, hyperinflation, and atelectasis. Pleural thickening and adhesions have been described in a minor proportion of patients as a result of chronic inflammation and recurrent exacerbation.^22^

### Computed tomography

CT, and high-resolution CT (HRCT) particularly, has radically changed the way by which the lungs can be viewed *in vivo* and is currently considered the most accurate non-invasive imaging modality for diagnosing numerous lung diseases, including bronchiectasis.^16^ The diagnosis of bronchiectasis, however, remains quite challenging in clinical practice. The relative ease of assessing severe bronchial dilation, observed in cystic bronchiectasis, strongly contrasts with the difficulty of depicting subtle earlier structural changes of cylindric bronchiectasis.^13,25^

#### CT protocols

There are no standardised HRCT protocols for the evaluation of bronchiectasis.^18^ For evaluating the airways, volumetric CT acquisition should be preferred, since it allows a precise assessment of the continuity of bronchial structures, while multiplanar reconstructions help differentiate bronchiectasis from cystic lung disorders (*e.g.* pulmonary Langerhans cell histiocytosis; lymphangioleiomyomatosis).^26^ According to current recommendations, images should be reconstructed with a slice thickness ≤1 mm to avoid overestimation of bronchial wall thickness and preferably assessed at a window level of −450 Hounsfield Unit (HU) - higher levels were demonstrated to increase the airway–artery ratio. Moreover, reconstruction kernels ought to be standardised for a more accurate longitudinal evaluation of bronchiectasis.^16,18,27,28^ Technical details are reported in Table 1.

**Table 1.** HRCT technical parameters for the assessment of airways (* kVp: Peak kilovoltage; **mAs: Milliampere seconds; ^#^ HU: Hounsfield unit)

Parameter	Value
Volumetric acquisition	-
Tube potential	120–80 kVp^*^; 100 or 80 kVp^*^ to be preferred for small size patients
Tube current	≤240 milliampere (mA);≤100 mAs^**^ or effective mAs^**^
Slice thickness	≤1.5 millimetres (mm)
Gantry rotation time	As short as possible (*e.g.* 200–500 ms), always ≤1 sec
Pitch	1–1.5
Reconstruction algorithm	High spatial frequency
Lung parenchyma windows mean/width	−400 to −700 HU^#^/>1000 HU ^#^
Soft tissue windows mean/width	50/350 HU

The use of iodine contrast is triggered by haemoptysis, a well-known and potentially life-threatening complication associated with severe bronchiectasis.^29^ Haemoptysis is usually caused by the rupture of a bronchial or pulmonary artery into the bronchial lumen resulting from bronchial artery dilatation and neovascularity due to recurrent airway inflammation, whereas bleeding from a bronchial vein is rare.^30–32^ Angiographic CT is the established diagnostic imaging modality to identify sources of haemoptysis and for preprocedural planning,^33^ whereas the evaluation of pulmonary arterial enlargement, an indirect sign of pulmonary hypertension that has been found to be a significant prognostic marker in patients with bronchiectasis, does not require intravenous contrast material.^34^

It is worth emphasising that the calibre of both the airway and the accompanying pulmonary artery branch depends on lung volumes at the time of scan acquisition.^35^ Lung volume standardisation should be pursued to increase both objectiveness and reproducibility of radiological interpretation of bronchiectasis, whose definition is mostly based on the assessment of the airway–artery ratio.^18^ Spirometer-controlled HRCT acquisition appears to be the optimal strategy to ensure adequate lung volumes and has been successfully used in some centres, mostly in children.^36,37^ The application of such acquisition method, however, remains limited in clinical practice,^17^ requiring patients to be highly cooperative and several professional figures to be involved at the time of acquisition.

Of note, differences in lung volume between baseline and follow-up CT scans might cause bronchiectasis to “disappear”.^18^ The concept of “reversible bronchiectasis”, originally described in bronchography and then in few case reports^38–40^ and retrospective studies employing CT,^41,42^ refers to a reversible bronchial dilation resulting from infection, inflammation or obstruction.^40,43^ Underlying mechanisms such as increased intraluminal pressure due to retained secretions, negative pressure secondary to atelectasis, and ineffective cough^44,45^ can lead to temporary airway dilation persisting up to months after the acute setting.^46^ Most often described in patients with pneumonia,^40,47^ these phenomena demand cautious evaluation of bronchial dilatation, particularly in consolidated or atelectatic lung areas, to avoid misinterpreting potentially reversible airway dilatation with bronchiectasis. Imaging follow-up at an appropriate time interval is of value to avoid this pitfall, as it enables differentiating bronchial dilation that returns to normal over time from cases in which irreversible destructive changes in the musculoelastic tissues of the bronchial wall have occurred.^45,48^

#### CT features of bronchiectasis

The most used criterion to determine the presence of bronchiectasis is the increased ratio of the cross-sectional diameter of an airway and its adjacent artery (airway–artery, AA, ratio).^13,16^ Such diagnostic criterion is affected by a number of limitations. First, the cut-off values for the AA ratio vary among different studies, and no reference interval has yet been validated.^49,50^ Nevertheless, 1.0 to 1.5 represents the interval ratio most frequently used in the literature. Furthermore, cut-off values should be age- and sex-dependent, as the AA ratio tends to increase in older subjects and decrease in infants.^51–53^ An AA ratio >0.8 has recently been suggested to define bronchiectasis in children and adolescents, and a ratio >1–1.5 in adults.^54^

No consensus exists on whether the either inner or outer airway diameter ought to be used to compute this ratio. If the inner diameter is used, the rate of false-negative diagnosis of bronchiectasis may increase, for instance, in case of mucus attached to the airway wall folding of the mucosa or when the CT is acquired at lung volumes below total lung capacity. These conditions can reduce the internal bronchial diameter andthus, modify the AA ratio.^55,56^ Likewise, all conditions that affect arterial diameter limit the reliability of the AA ratio. The so-called hypoxic pulmonary vasoconstriction, an adaptive mechanism in which alveolar hypoxia causes local pulmonary vasoconstriction, can increase the AA ratio and mimic bronchiectasis. Such a condition may be secondary to smoking and high altitude.^12,57^ Conversely, pseudonormalisation of the AA ratio may occur in case of pulmonary arterial enlargement.^24^

The other two most used criteria to diagnose bronchiectasis are represented by lack of tapering, defined as unchanged airway diameter for 2 cm after branching, and visualisation of airways in the periphery of the lung, within 1 cm from the costal pleura or abutting mediastinal pleural.^58,59^ These two criteria seem more reliable than the AA ratio, though their visual assessment is still limited by some degree of subjectivity.^17^

Other CT findings commonly associated with bronchiectasis include bronchial wall thickening, airway plugging, mosaic attenuation, and volume loss. Bronchial wall thickening usually represents airway inflammation.^13^ However, the definition of the underlying cause of bronchial wall thickness is rather difficult; stasis, mucus, and longstanding structural changes caused by repeated cycles of injury and repair (*i.e.* the process of remodelling) can result in bronchial wall thickening and lead to different degrees of functional impairment.^58,60,61^ Notably, evaluation of bronchial wall thickness is largely subjective in everyday practice and thus, increases variability in differentiating normal from abnormal (*e.g.* in cases of slight, even diffuse, thickness increase). Even in patients with advanced bronchiectasis, visual subscoring systems of bronchial wall thickness have shown intra- and inter-reader agreement values ranging from moderate (intraclass correlation coefficient, ICC = 0.5–0.56) to good (ICC = 0.67–0.73), respectively.^62–64^

**Figure 1.**
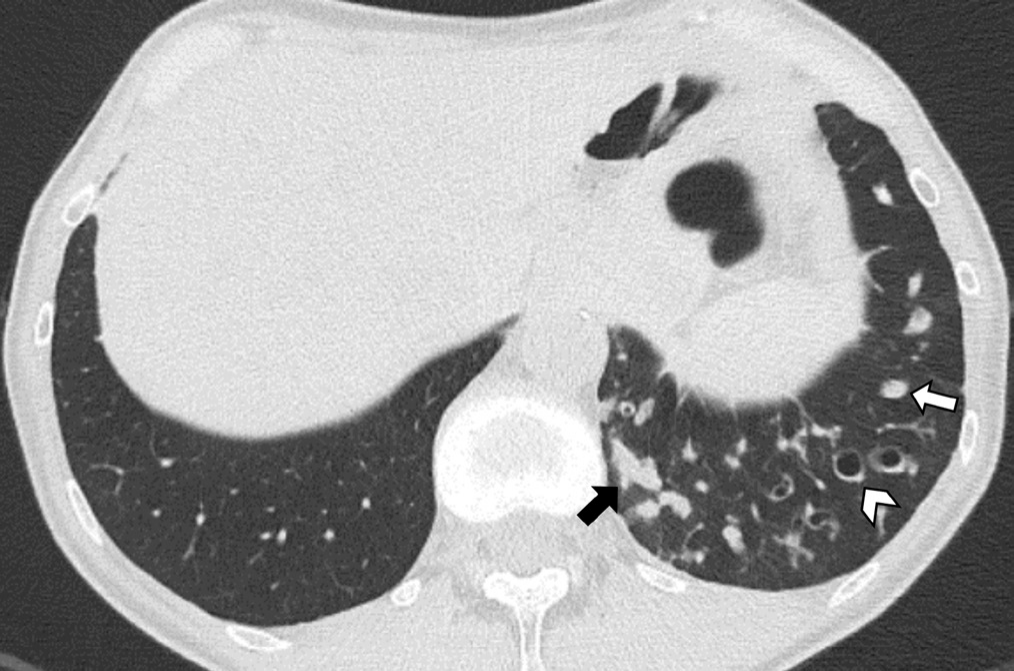
Axial CT image shows cylindrical bronchiectasis in the left lower lobe. The disproportion between the bronchi and the corresponding pulmonary arteries running perpendicular to the axial plane recalls the “signet ring” appearance (arrowhead). Mucus plugging appears as tubular (black arrow) or nodular (white arrow) opacities depending on the bronchial orientation relative to the scan plane.

**Figure 2.**
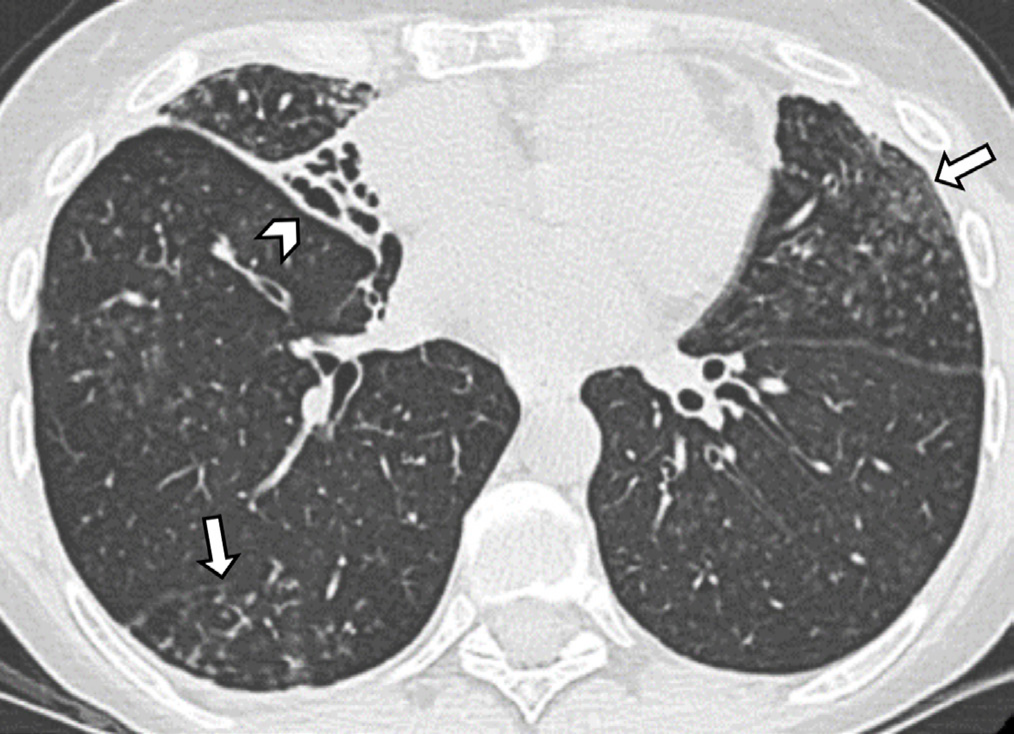
Axial CT image in a patient diagnosed with non-tuberculous mycobacterial infection shows varicoid bronchiectasis and volume loss in the middle lobe (arrowhead). Bilateral centrilobular nodules and tree-in-bud opacities (arrows) due to distal airways involvement can be appreciated in the right lower lobe and in the left upper lobe.

Airway plugging appearance varies according to the length and orientation of the abnormal airway relative to the scan plane. Filled dilated bronchi will manifest as tubular, Y-, or V-shaped opacities when seen along their long axis, while resulting in “nodules” or “dots” if perpendicular to the image plane. In this latter case, the tree-in-bud pattern is observed when mucus secretions fill distal airways^65,66^ (Figures 1 and 2).

**Figure 3.**
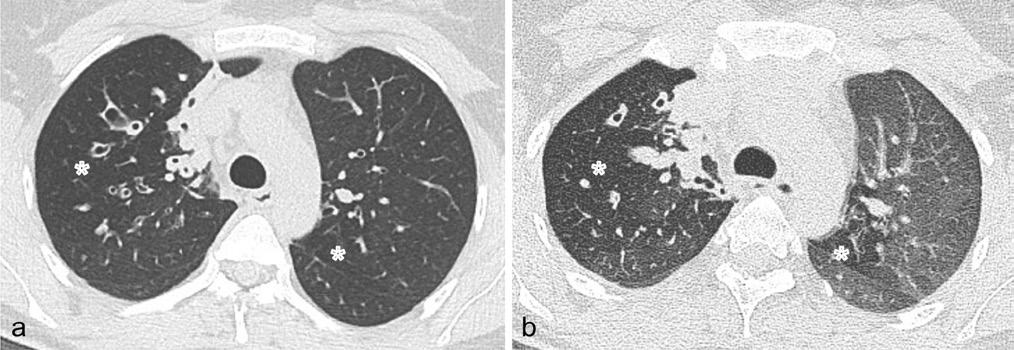
(a) Inspiratory axial CT image shows thick-walled bronchiectasis within areas of slightly decreased attenuation (asterisks) in the upper lobes. (b) Expiratory axial CT image acquired at the same level showed no changes in density of the hypo-attenuated areas (asterisks), demonstrating the presence of air trapping.

Small airways involvement is regarded as an integral part of bronchiectasis.^67^ Air trapping is almost invariably associated with bronchiectasis, even in lobes without overt bronchiectasis, suggesting that obliterative bronchiolitis may be an early event in the pathogenesis of the disease. It was postulated that constrictive obliterative bronchiolitis might represent the first event that leads to proximal bronchial dilatation over time.^68^ Therefore, additional expiratory CT scanning is helpful in the presence of bronchiectasis to improve the interpretation of the mosaic attenuation pattern (i.*e.* to differentiate between air trapping and ground glass opacification)^69^ (Figure 3).

Both signs of volume loss (*e.g.* bands of atelectasis, displacement of the fissures, etc.) and crowding of the airways are due to peribronchial inflammation and fibrosis. (Figure 2). Such distorted bronchiectasis should not be defined as traction bronchiectasis, a term that refers to airways irregularly dilated within CT features of lung fibrosis, such as peripheral reticular opacities and honeycombing.^24^

**Figure 4.**
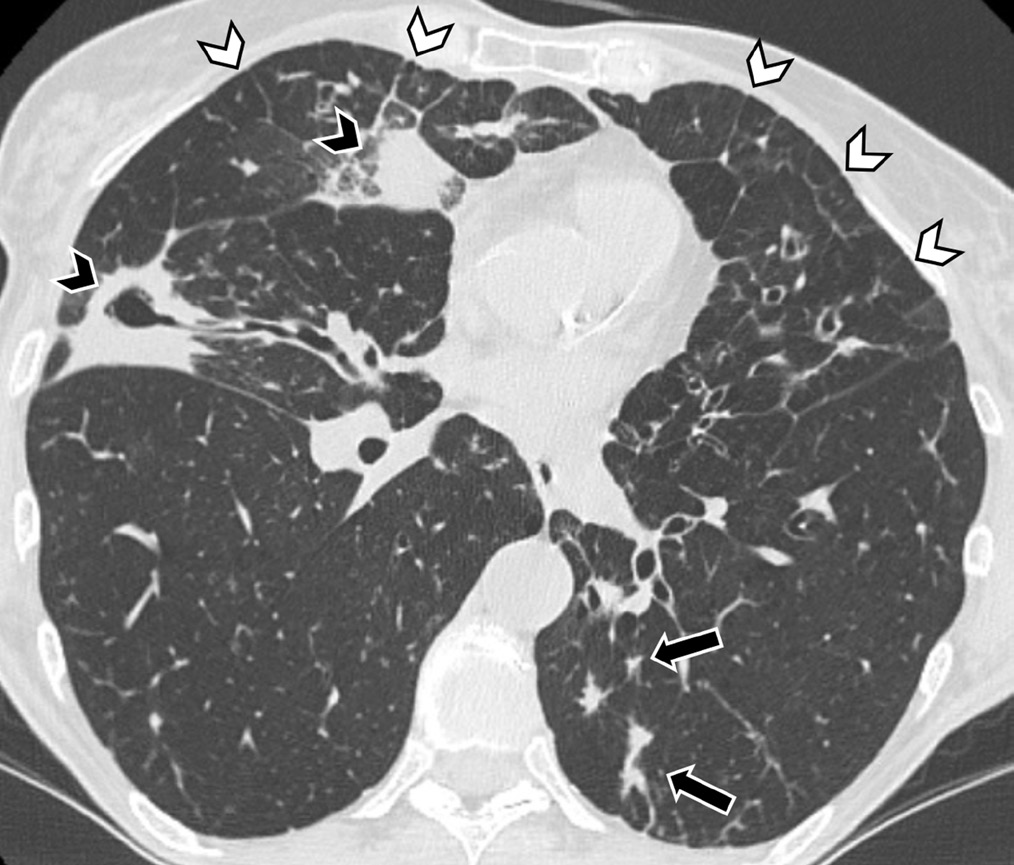
Axial CT image shows extensive bilateral thick-walled bronchiectasis with mucus plugging (black arrows), consolidation (black arrowheads) and thickened interlobular septa (white arrowheads).

Thickening of interlobular septa has been described in a relevant proportion of patients diagnosed with idiopathic bronchiectasis (Figure 4). It was suggested that interlobular thickening might be secondary to septa infiltration by inflammatory cells or lymphatic congestion (*e.g.* due to increased or obstructed lymphatic flow). Notably, the extent of bronchiectasis correlated to the profusion of thickened interlobular septa.^70^

## Severity of bronchiectasis: radiological scores

Over the decades, several radiological scoring systems have been proposed to quantify disease severity.

In 1991, Bhalla et al proposed the first CT-based scoring system, which allows a comprehensive characterisation of bronchiectasis, encompassing nine different features, also including the presence of emphysema.^19^ Although the Bhalla score was developed on only 14 CF patients, it was largely applicated to non-CF bronchiectasis in both clinical and research settings.

Subsequently, a simplified scoring system – the Reiff score – was derived from the Bhalla score. The Reiff score, whereby each lobe is assessed separately, was developed on a significantly larger number of patients (146), including both CF and non-CF ones.^20^

The more recent Bronchiectasis Radiologically Indexed CT Score (BRICS) was also derived from the Bhalla score but was developed in a specific cohort of non-CF patients: affected by either idiopathic or post-infective bronchiectasis with limited smoking history. Of note, this score is the only one that has been externally validated, showing consistent results in a validation cohort of more than 300 patients from 6 different European centres. The BRICS CT metrics of bronchiectasis were derived from multivariable models predictive of clinical disease severity markers. Interestingly, the multivariable models retained two CT features: bronchial dilation and number of bronchopulmonary segments affected by emphysema. These two combined CT features showed a significant correlation with clinicoprognostic markers, making the BRICS score an attractive easy-to-use method to assess non-CF bronchiectasis in routine practice.^21^

Nevertheless, these CT-based scoring systems have several limitations. Firstly, the Bhalla score from which the others were derived was developed for patients with CF, though applied to all types of bronchiectasis.^71^ Secondly, they all rely on a subjective judgement of the severity and extent of specific features of bronchiectasis (*e.g.* bronchial dilation, bronchial wall thickening and mucous plugging) and still do not capture the heterogeneity of the disease. For example, patients with structurally more abnormal but localised disease can be scored as high as those with widespread but less prominent structural abnormalities.

A substantial weakness of these scoring systems is represented by the lack of integration with clinical parameters. The BRICS takes into account clinicoprognostic aspects but fails to correlate specific CT features with the degree of disease activity, which influences treatment choice.^1^ Multidimensional scores, such as the Bronchiectasis Severity Index (BSI), and the FEV1, Age, Chronic colonisation, Extension and Dyspnoea (FACED) score, have attempted to integrate clinical and prognostic parameters with CT features of bronchiectasis,^72,73^ but include a rather limited number of clinical features, albeit relevant for the majority of the affected patients, potentially irrelevant for others. Moreover, these systems could not overcome the intrinsic limitations of the current CT criteria (*i.e.* the AA ratio measurement).

In this heterogenous clinical and radiological context, it is unlikely that a single scoring system would be suitable for all types of bronchiectasis, which does not represent a definite pathological entity, but rather a complex spectrum of disorders.

### Confident radiological diagnosis of bronchiectasis

Although CT findings are usually of limited value to discriminate among different causes of bronchiectasis,^20^ it is worth reiterating that the type of bronchiectasis (*i.e.* cylindric, varicose or cystic), their distribution within the lung regions and concurrent abnormalities might help narrow the differential diagnoses.^24,74^ Among others, associated pathological features include abnormal tracheal dilatation, mucous plugging, tree-in-bud nodular pattern, consolidation, and atelectasis. Visual interpretation of CT images should entail the assessment of the location and spatial distribution of bronchiectasis (*i.e.* apical *vs* basal and central *vs* peripheral), their extent (*i.e.* focal *vs* diffuse), morphology (*i.e.* cylindric, varicose or cystic), and severity.^75,76^ Focal bronchiectasis, for instance, is frequently of acquired origin (*e.g.* extrinsic compression, endobronchial malignancies, foreign body aspiration) and is far less common than diffuse bronchiectasis.^75^ Bronchiectasis with upper and mid lobes predominance are typical of CF, sarcoidosis and non-tuberculous mycobacterial infections; a central predominance is more commonly encountered in ABPA, while a lower lobes predominance is characteristic of chronic aspiration or much rarer pathologies, such as primary ciliary dyskinesia (PCD) and congenital immunodeficiency.^24,25,75^

Non-CF bronchiectases recognise numerous causes, but there are only a handful of conditions whereby CT features are highly suggestive of a specific underlying cause. These conditions that share a low or extremely low prevalence are briefly discussed below. The main causes of non-CF bronchiectasis and associated CT features are reported in Table 2.

**Table 2.** Main causes of non-CF bronchiectasis and associated CT features

Causes	Type of bronchiectasis	Distribution	Associated and/or distinctive features
*Congenital*			
Mounier-Kuhn Syndrome^77^	NA (Trachea and main bronchi)	Central lung regions	Bronchial diverticulosis, tracheal diverticula
Williams-Campbell Syndrome^78^	Varicose, cystic	Sub segmental bronchi (fourth to sixth generations)	Collapsed bronchi and distal air-trapping on expiratory CT
α 1-Antitrypsin deficiency^79^	Cylindric, cystic	Mainly lower lobe	Panlobular emphysema
*Immunologic*			
Allergic bronchopulmonary aspergillosis^16^	Cylindric, varicose	Segmental and subsegmental bronchi of central-upper lungs regions	Mucous plugging (“finger-in-glove” sign)
*Infectious or inflammatory*			
Bacterial, mycobacterial, viral	Various	Various	Depend on pathogens
Swyer-James Syndrome^80^	Cylindric	Non-specific	Hyperlucent lobe or lung and air-trapping
Chronic aspiration^80^	Cylindric	Basal lung regions	Bronchial wall thickening, tree-in-bud consolidations
*Defective mucous transport*			
Primary Ciliary Dyskinesia^81^	Varicose, cylindric	Middle and lower lobes	Situs inversus
Young’s Syndrome^82^	Cystic	Little evidence	Little evidence
Primary immunodeficiency^83^	Mainly cylindric	Upper and mid lung regions	Non-specific
*Airways obstruction*			
Endobronchial malignancies^75^	Various	Focal	Various
Broncholithiasis^80^	Cylindric, varicose	Focal, more often middle lobe	Calcified lymph nodes
Extrinsic compression^75^	Various	Focal	Various
*Idiopathic*	Various	Basal lung regions^20^	Various

### Allergic bronchopulmonary aspergillosis (ABPA)

ABPA is a disorder characterised by chronic inflammation and airways damage due to persistent colonisation and sensitisation by Aspergillus species. It typically affects patients with asthma (up to 14% of corticosteroid-dependent asthmatic patients) and CF (6%).^84^ Radiological findings are quite specific and consist of bronchiectasis with a central-upper lungs predominance, bronchial wall thickening, mucoid impaction that frequently results in tubular branching opacities – the so-called “finger-in-glove” sign – and high attenuation mucus plugs (probably due to the contents of calcium). The recognition of these CT features allows a confident diagnosis in most cases in which ABPA is suspected.^16,24^

### Tracheobronchomegaly (Mounier-Kuhn syndrome)

**Figure 5.**
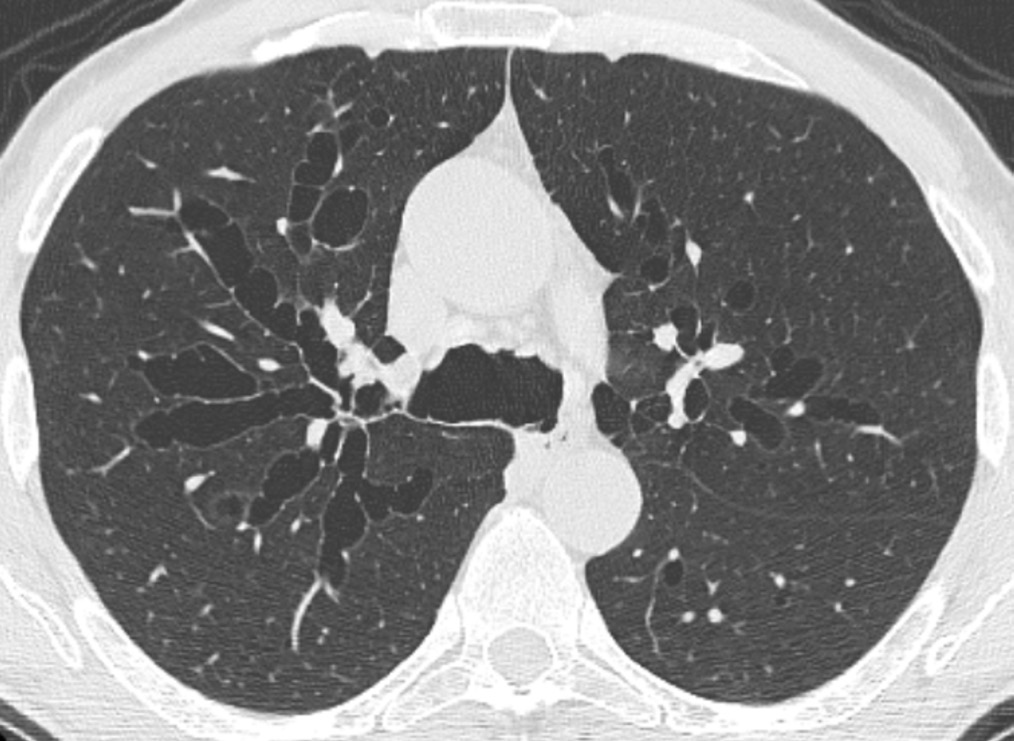
Axial CT image demonstrates enlarged main stem bronchi and thin-walled central bronchiectasis consistent with Mounier-Kuhn syndrome.

Tracheobronchomegaly, also known as “Mounier-Kuhn syndrome” is an extremely rare disorder of unknown prevalence characterised by an abnormal dilatation of the trachea and major bronchi. Most cases are congenital but acquired forms have been described in association with other disorders, such as pulmonary fibrosis.^85^ CT usually permits an accurate diagnosis of the disease, showing the marked increase of the calibre of the tracheobronchial tree, with a characteristic corrugated appearance of its walls^77^ (Figure 5).

### Williams-Campbell syndrome

Williams-Campbell syndrome is a rare disease characterised by a deficiency of cartilaginous tissue in subsegmental bronchi. CT findings consist of cystic bronchiectasis with thickened walls involving bronchi from the fourth to the sixth generations. Expiratory CT scan shows the collapse of cystic dilated bronchi, one of the most characteristic signs of such syndrome. These findings are quite peculiar, and their identification makes the diagnosis quite straightforward.^78^

### Primary ciliary dyskinesia (PCD)

**Figure 6.**
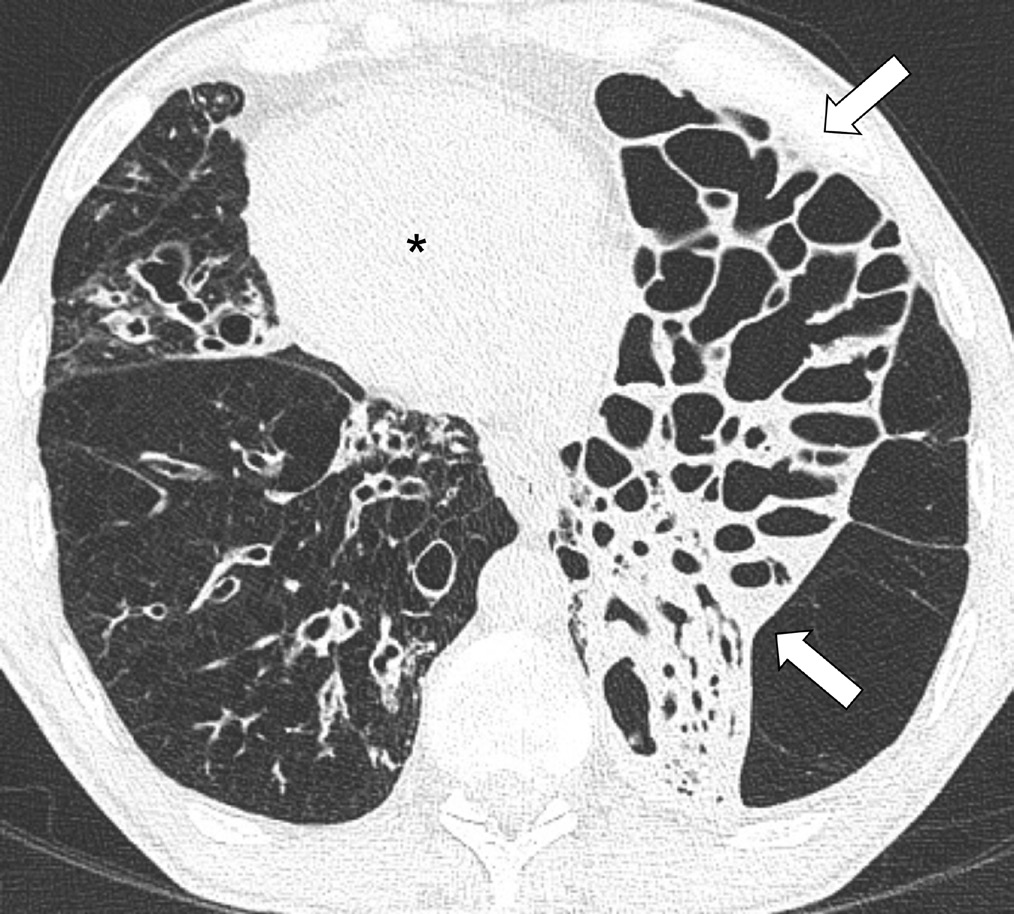
Axial CT image of a patient diagnosed with Kartagener syndrome shows dextrocardia (asterisk) and bilateral bronchiectases involving the middle lobe and both lower lobes. Marked volume loss is evident in the left lower lobe (arrows).

PCD accounts for up to 8% of adults non-CF bronchiectasis. In this inherited disorder, ultrastructural defects of the ciliary apparatus result in abnormal or absent beating of cilia.^86^ CT typically demonstrates the presence of varicose bronchiectasis, predominantly distributed in the middle and lower lung lobes and associated with atelectasis, tree-in-bud nodular consolidation, and mucous plugging.^81^ Moreover, half of the patients have Kartagener syndrome, which is defined by the triad of bronchiectasis, situs inversus totalis, and either nasal polyps or recurrent sinusitis^86^ (Figure 6). In such cases, the diagnosis of PCD-associated bronchiectasis is easily and confidently performed.

### Longitudinal evaluation of bronchiectasis

High-quality evidence in favour of repeated imaging in patients with bronchiectasis still lacks. Structural changes underlying fluctuations in pulmonary function such as the degree of bronchial wall thickening and mucous retention are not necessarily evident on CXR, making correlations of expected radiographic abnormalities with clinical and functional features of disease worsening of limited reliability.^87,88^ Repeated HRCT has otherwise shown promise for assessing physiologically relevant pulmonary changes, but it carries radiation risk and should be managed with caution.^89^ Current indications for repeated HRCT include chronic and acute clinical deterioration.^54,87^ According to the updated British Thoracic Society guidelines, in fact, a deteriorating patient should be assessed with chest CT and administration of iodine contrast ought to be considered when pulmonary embolism is suspected. Clinical deterioration is defined by significant and prolonged worsening of symptoms, rapid decline in lung function, increased frequency or severity of exacerbations, frequent hospital admissions and/or early relapse after treatment of an exacerbation episode.^87^ It is worth considering that patients who experience a slowly progressive clinical decline do not necessarily display the same CT features as those who present with acute clinical deterioration. For instance, morphological features of active disease, including increased bronchial wall thickening, mucous plugging with atelectasis, and parenchymal consolidation, are more likely to be depicted in acutely deteriorating patients, where the mere assessment of bronchial dilatation severity is of limited utility.

Regardless of the specific clinical setting, which has to be taken into account, establishing a radiological progression of bronchiectasis is quite difficult. Definite criteria still lack, and the limitations of current radiological scoring systems remain.^90^ To date, most studies that investigated the radiological evolution of bronchiectasis were performed on CF patients.^89,91^ Park et al, conversely, only enrolled non-CF patients, demonstrating that lower body mass index and isolation of *Pseudomonas aeruginosa* in respiratory specimen are associated with radiological progression of bronchiectasis.^92^ Such progression, however, was assessed using the Bhalla score, whose weaknesses have already been highlighted.

### Quantitative imaging and future directions

Despite the large and consistent body of literature suggesting a prognostic role for HRCT in bronchiectasis, HRCT-based biomarkers are neither routinely used in clinical practice nor encompassed as a clinical end point in therapeutic trials. Quantitative evaluation of bronchiectasis suffers from a limited capability to reflect disease heterogeneity and activity, resulting in discrepancies between the radiological disease severity and prognostic information.^93^ The latter is particularly relevant since patients at a higher risk of recurrent exacerbations might benefit from more aggressive treatments, which are not free of risks.^56^ Moreover, visual assessment of HRCT findings and their extent has shown significant interobserver variation, even among expert radiologists, affecting both baseline and follow-up evaluation.^94–96^

Automated and semi-automated algorithms have the potential to overcome the limitations of the time-consuming manual annotations and visual scoring methods to quantify abnormal widening and thickening of airways. Significant differences in several parameters between subjects with bronchiectasis and controls have been described in the literature.^56,97,98^ These include either measurements that use artery size for normalisation or depend on the airway size only (*i.e.* wall thickness and lumen) or tapering. Their implementation in a clinical setting is still limited due to technical issues inherent to CT or related to algorithms’ performances: slight differences in attenuation values that affect the differentiation of wall thickening from mucous obstruction or peribronchial abnormalities; airways mislabelling; limited capability of extracting branches and pairing artery.^99^ Moreover, studies evaluating automatic and semi-automatic airways extraction in patients with bronchiectasis lack generalisability due to small and non-randomised study populations, lack of ground truth, and non-standardised protocols.^62,97,98^ Current evidence in supporting density-based CT scoring methods, a possible biomarker that does not rely on the complex workflow of airway extraction, mostly derives from the clinical setting of CF and suffers from similar limitations.^100,101^

Artificial intelligence has shown encouraging results in other fields of thoracic imaging,^101^ but if and how it will play a role in bronchiectasis remains to be determined. Learning techniques, such as convolutional neural networks, may have the potential to support radiologists in approaching bronchiectasis systematically, possibly minimising bias of subjective evaluation.^102^ Ideally, future studies should capture several aspects of the disease, including genomic, metabolomic, and clinical information, resulting in highly complex data sets to be mined with artificial intelligence to gain new knowledge regarding the diagnosis, classification, and treatment of bronchiectasis.
